# Increased creatine demand during pregnancy in Arginine: Glycine Amidino-Transferase deficiency: a case report

**DOI:** 10.1186/s12884-020-03192-4

**Published:** 2020-09-03

**Authors:** Maria Grazia Alessandrì, Francesca Strigini, Giovanni Cioni, Roberta Battini

**Affiliations:** 1 Department of Developmental Neuroscience, IRCCS Stella Maris Foundation, Via dei Giacinti 2, 56128 Calambrone - Pisa, Italy; 2 Department of Clinical and Experimental Medicine, University of Pisa, Pisa, Italy

**Keywords:** Creatine deficiency, AGAT, Pregnancy

## Abstract

**Background:**

Creatine (Cr), an amino acid derivative, is one of the most important sources of energy acting as both a spatial and temporal energy buffer through its phosphorylated analogue phosphocreatine (PCr) and creatine kinase (CK). Maternal Cr biosynthesis and metabolism seem to play an important role in pregnancy, as shown in preclinical and in healthy human pregnancy studies. Patients with Arginine:Glycine Amidino-Transferase deficiency (AGAT-d), due to the deficit of the first enzyme involved in Cr synthesis, are at a disadvantage due to their failure to synthesize Cr and their dependence on external intake, in contrast to normal subjects, where changes in Cr biosynthesis supply their needs.

We report the outcomes of a pregnancy in an AGAT-d woman, and the challenge we faced in managing her treatment with oral Cr to ensure optimal conditions for her fetus.

**Case presentation:**

A 22-year-old AGAT-d woman referred to our Institute for the management of her first conception at 11 weeks of fetal gestational age. Sonographic monitoring at 20 w GA indicated a reduction of fetal growth, in particular of the head circumference that was below the 3^rd^ centile. Biochemical monitoring of Cr in biological fluids of the mother revealed a decline of the Cr concentrations, in particular in the urine sample, requiring prompt correction of the Cr dose. At 35 weeks of gestation the patient delivered a male infant, heterozygous for *GATM* mutation, with normal brain Cr levels; at one year the baby achieved typical developmental milestones.

**Conclusions:**

This rare pregnancy demonstrates that Cr levels in the blood and urine of the mother with AGAT-d decreased since the first months of gestation. The increase of the Cr daily dose administered to the mother seems to have produced beneficial effects also on the fetus.

## 1. Background

Cr is synthesized in a two-step reaction, where the rate-limiting step is the Arginine: Glycine Amidino-Transferase (AGAT) reaction to form guanidinoacetic acid (GAA) from arginine and glycine; GAA is quickly converted to Cr by guanidinoacetate methyltransferase (GAMT). AGAT deficiency (AGAT-d; MIM 602,360) causes the rarest inborn error of Cr deficiency syndromes [1, 2]. Chronic Cr supplementation at various dosages, ranging from 100 mg/Kg/day to 800 mg/Kg/day, restored brain Cr in all patients diagnosed so far and improved cognitive development and other clinical symptoms [2].

Cr, an amino acid derivative, is one of the most important sources of energy due to its primary function as both a spatial and temporal energy buffer. By way of the phosphorylated analogue phosphocreatine (PCr) and creatine kinase (CK) it transfers high energy groups from mitochondria to sites of consumption and regenerates ATP from ADP.

Maternal Cr biosynthesis and metabolism seem to be critical in pregnancy [3, 4], as fetal growth requires increased energy requirements, metabolic adaptations and specific nutritional needs for energy accrual. The state of the art literature on the topic currently includes preclinical studies and ongoing work in healthy human pregnancies. Recently, it has been demonstrated that the human placental homogenates are able to synthesize Cr [5] in addition to transferring amino acids, glucose, and oxygen to the fetus. Maternal Cr levels have been associated with fetal growth in a large cohort of healthy women, as reported by Dickinson and colleagues [4]. Furthermore, an increased demand for Cr during pregnancy was described in the spiny mouse animal model [3]. Therefore, it is conceivable that pregnant AGAT-d women are more susceptible to a Cr deficit due to energy demands of the fetus. In the AGAT deficiency mouse model [6] the homozygous knockout mice were unable to reproduce and to date no children from patients with AGAT deficiency have been reported.

This case report presents, for the first time, the pregnancy of a woman with AGAT-d and the challenges posed in the management of her treatment with oral Cr to ensure optimal conditions for both the mother and her fetus. The clinical management was accomplished by correcting the maternal Cr intake according to clinical and biochemical evidence.

## 2. Case Presentation

### Patient

A 22-year-old AGAT-d patient informed us about her pregnancy at 11 weeks of fetal gestational age (GA), confirmed by earlier ultrasonographic examination (at 6 and 11 weeks GA).

At the age of six a diagnosis showed she was carrying a homozygous mutation in the GATM gene [c.446 g > A, p.(W149X)] [7] and since then had been administered high doses of Cr monohydrate dissolved in water (Galeno srl, Italy) with successful management results. Regular and long-term follow up had been maintained over the years by monitoring urinary, plasma and brain Cr levels [8]. Briefly, blood and morning urine samples were obtained after an overnight fasting period before taking the first dose of Cr. To analyze plasma samples, the blood collected in EDTA tubes was centrifuged for 15 min at 1800 rcf and the plasma separated; 200 µl of plasma or urine were analyzed as described [9].

In the last examination 10 months before her pregnancy, plasma and urinary Cr values were 59.4 µmol/L (normal values: 18–141 µmol/L) and 789.6 µmol/L (normal values: 200–5500 µmol/L), respectively, and the daily Cr dose was 2 g; brain Cr was stable around 90% of normal. However, through the long-term follow-up of the patient, we observed considerable fluctuations in Cr concentrations reflecting her low compliance and self-adjustment of her daily dose in order to lose weight. Her weight at the beginning of pregnancy was of 78 kg with a BMI of 26.67 (weight/h^2^). The patient was omnivorous with great preference for meat-rich foods.

### Diagnostic assessment and therapeutic intervention

During pregnancy, the patient underwent routine laboratory and sonographic tests as recommended by typical pregnancy guidelines. The first examination for urinary and plasma Cr concentrations was at 11 + 5 weeks + days (w + d) of GA; subsequent examinations were scheduled at 15, 20, 27 and 36 weeks of gestation, with the possibility of reducing the interval between examinations, if necessary. Samples were sent by courier to our laboratory within 24 h of collection, kept on ice, and analysed on the same day of delivery. Analyses of urinary and plasma Cr were performed by GC/MS [9]; the analysis of creatinine (Crn) was performed by enzymatic assay (Sentinel Diagnostics, Italy).

The patient was advised to strictly adhere to the Cr supplementation at prescribed dosages (2 g/day).

*GATM* sequencing of her husband found that he was homozygous for the normal alleles of the GATM gene.

### Follow-up and treatment

Routine analyses results were all within the normal range for pregnancy (full blood count, liver and renal function, glucose load test); in particular, glycemia ranged from 88 to 81 mg/dl and no gestational hypertension was reported. Her body weight gain throughout the pregnancy was 20 kg and at delivery she weighed 98 kg with a BMI of 33.5 (weight/h^2^).

Plasma Cr concentrations slowly decreased during the first weeks of gestation, ranging from 66.8 µmol/L at 11 + 5 (w + d) to 53.1 µmol/L at 15 weeks GA. Conversely, urinary Cr at that time was 279.5 µmol/L, about one third of the value recorded at preconception (789.6 µmol/L). Routine ultrasound control at 20 w GA highlighted reduced fetal brain development as indicated by head circumference (HC) value of 176.2 mm (< 2.3 centile) [10]. At 20 + 3 (w + d) GA a further decline in plasma Cr concentrations with value of 30.4 µmol/L was observed (Table 1); urinary Cr value was 286.4 µmol/L. To avoid the risk of microcephaly, the Cr dose was promptly increased to 3 g /day; at 21 + 3 (w + d) GA an increase of plasma Cr level was observed (45.5 µmol/L), while urinary Cr value still remained low (297.9 µmol/L). After 5 weeks an increase of Cr levels in both plasma and urine (76.2 and 5533 µmol/L, respectively) were observed; the fetal head growth did not show any further decrease in size as compared to the normal range for GA, but rather showed an increasing trend (HC 234.6 mm, 3rd centile). During the last biochemical examination before delivery, at 34 + 5 (w + d) GA, a further increase of Cr concentration was detected (Table 1). At that time, urinary Cr and Crn values rose markedly to 10,806 µmol/L and 17.1 mmol/L, respectively (Table 1). Plasma and urine GAA concentrations in the mother were always under the normal values or undetectable.

**Table 1:** Fetal parameters and maternal biochemical parameters

GA	CRL	BPD	HC	AC	FL	Maternal plasma Cr	Maternal urine Cr	Maternal urine Crn	Maternal Cr dose
**w + d**	mm (centiles)	mm (centiles)	mm (centiles)	mm (centiles)	mm (centiles)	µmol/L n.v.: 18–141	µmol/L n.v.: 200–5500	mmol/L	g/day
**0**	-	-	-	-	-	59.4	789.6	7.1	2
**11 + 5**	50.8 (35)	-	-	-	-	66.8	678.2	12.2	2
**20 + 3**		47.7 (16)	176.2 (< 2.3)	148.4 (13)	32.5 (16)	30.4	286.4	6.1	2
**26 + 5**		66.1 (37)	234.6 (3)	208.6 (10)	48.7 (25)	76.2	5533	3.7	3
**34 + 5**						85.0	10,806	17.1	3

*GA (W + d)* Gestational age (Weeks + days), *CRL* crown rump length, *BPD *biparietal diameter, *HC *Head circumference, *AC *abdominal circumference, *FL *femoral length, *Cr *creatine, *Crn* creatinine, *n.v. *normal values. In the brackets the centiles for the fetus growth

At 35 weeks the patient delivered a male newborn by planned caesarean section, weighing 2450 g (25th centile), with a length of 49 cm (25th centile) and HC of 32 cm (10th centile); APGAR score was 8–9. The newborn plasma and urinary concentrations of Cr were 56.4 and 103 µmol/L, respectively. The guanidinoacetic acid (GAA) values in the infant plasma and urine were both low (0.15 and 17.2 µmol/L, respectively; GAA reference range: 0.22–3.14 µmol/L in plasma, 56–698 µmol/L in urine, respectively). He was breastfed for 4 months during which the mother continued taking 3 g/day of Cr; at that time the plasma Cr of the child was 49.4 µmol/L, the Cr on brain MRS was normal as well as brain MRI.

The boy reached normal developmental milestones; his weight and height remained at 25th centiles. At four months his sucking was normal as were his muscle tone and strength; the general movements examination (GMs) was also normal (present fidgety movements). At 12 months of age, the plasma and urinary Cr of the baby increased to 67.7 and 369.7 µmol/L; the plasma and urinary GAA concentrations were 0.56 µmol/L and 250.2 µmol/L, respectively. The child’s psychomotor development was normal (Bayley III) and auxological data, including HC, were at the 25th centile. After 4 months of breastfeeding, the mother, of her own accord, decided to reduce her Cr supplementation, returning to the dose prior to her pregnancy of 2 g daily, because of weight gain.

## 3. Discussion

This report, the first presenting the course of a pregnancy of a woman carrying a homozygous mutation in GATM gene who had always been fully dependent on an external Cr supply since an AGAT-d diagnosis at 6 years, provides additional evidence that Cr may be important during pregnancy. The most relevant findings were (i) decreased levels of urinary and, to a lesser extent, plasma Cr concentrations in the first months of fetal gestation; (ii) reduction of fetal HC below 2.3 centile detected at 20 weeks GA and (iii) partial recovery of auxological parameters of the fetus once the Cr dose administered to the mother had been increased.

The clinical data reported here must be carefully interpreted, because the observed results may have been biased by the study limitations. The limitations included full dependency of the mother on an external Cr supply, some difficulties in communication between medical staff and the patient, and the treatment of the patient at home due to the considerable distance between her home and our hospital.

Cr homeostasis during pregnancy changes in both animal models and in humans. Studies with spiny mice have shown decreased renal excretion between middle and late gestation, and modifications of the expression of synthetic enzymes and transporter [3]. In healthy women, Dickinson et al. [4] observed that plasma Cr concentrations were stable across gestation whereas urine levels decreased significantly at 18 weeks GA. By contrast, Pinto et al. [11] reported a decrease in both plasma Cr and Crn in the first trimester only, and no correlations between plasma and urinary Cr and Crn concentrations. In our patient, plasma Cr always remained within the normal range over the pregnancy, while urinary Cr concentrations quickly fell within a few weeks reaching the lowest normal values. The Cr decrease in plasma and more markedly in urine between 11 and 20 weeks may have been due to fetal demand, given the maternal compliance which was consistently monitored starting from the 11th week. Whether the decrease of Cr in body fluids is due to alterations in its distribution between plasma and tissues, or to a reduced absorption of Cr supply across the gut during gestation, or even to an increased fetal demand that drains Cr from the mother, is currently unknown. From this finding, urinary Cr concentrations in pregnant women could be considered a more suitable marker to monitor fetal requirements for his/her optimal growth. This is consistent with the observations of Dickinson et al. who reported a positive association of urinary Cr levels with birthweight and birth length [4].

Furthermore, Cr is crucial for brain development, concentrations of which gradually increase in a normal fetal brain during GA as energy needs and cerebral structures evolve and double at the end of pregnancy [12]. Recent studies demonstrate that normal human placental homogenates can endogenously synthesise Cr, and abundant AGAT protein was detected in the human placenta at term [5]. Moreover, the mRNA for the Cr transporter (SLC6A8) has been detected in the first trimester chorionic villus biopsies (CVBs) and in placental samples [13], suggesting that the active transport of Cr into placental cells is in place from early gestation. Based on this speculation, the Cr transporter seems to be essential for the development of the fetus, representing the only way to provide him with the energy necessary for development, at least until his AGAT and GAMT are properly functioning. Further studies in this area would be beneficial so as to acquire greater knowledge regarding the time at which the Cr synthesis is fully developed in the fetus and the contribution of placenta in providing adequate Cr supply for fetal development. Nevertheless, the inability of our patient to synthesize Cr sufficiently, the still unknown role of the fetal placental tissue (which may have the enzymes for synthesis) and the high energy demands of the developing fetus not balanced by the amount of Cr absorbed considering her daily meat intake, led us to increase the maternal Cr supply. Based on our previous experience in patients with AGAT-d, the Cr dose was empirically raised to 3 g/day after the test at 20 + 3 GA, followed by a parallel increase of plasma Cr after 1 week [8]. One week before delivery the concentrations of Cr and Crn in the patient increased further, without signs of renal toxicity. These high values were not surprising, because we found that the mother had had similarly high values after Cr supplementation at 200 and at 100 mg/Kg. When this occurred over the years the Cr dose was gradually reduced to 2 g/day while ensuring, at the same time, that both the plasma and brain Cr concentrations were within the normal range [8] and adverse effects were avoided. Even on those occasions however, her urinary Cr and Crn levels sometimes rose to similar high values. Then again, it was difficult to predict the ratio of Cr absorbed after the increased dosage because it could potentially vary during pregnancy. In addition, various other factors could have contributed to the increase of the Cr and Crn levels observed, such as reduced hydration, alterations in the renal function in the final months of pregnancy, changes in hormonal metabolism and dietary intake, and placental changes in Cr transfer near birth [13]. As the Cr levels increased in the biological fluids of the patient, some parameters of fetal growth increased or stabilised, with the only exception of abdominal circumference (AC,10th centile), which decreased over the same time period. In particular, the effects of the increase in the Cr dosage on auxological parameters of the fetus were quickly evident, as had already been observed in the mother at diagnosis a few months after starting the Cr supplementation [8].

The GAA levels in both the plasma and urine of the one day old newborn were detectable but below the normal range, showing that he was already able to synthesize Cr on his own, despite his heterozygous status for *GATM*, since GAA could not come from maternal supply due to the undetectable levels in her plasma. The GAA concentrations increased during his first year reaching normal values similar to those in adults heterozygous for AGAT-d, where the plasma concentrations of GAA are not dissimilar from those of the homozygous for the normal alleles of *GATM*. Conversely, plasma Cr levels were normal at 1 day of birth, presumably for the partial contribution of Cr from the mother, while the concentrations in urine were low in the first day of life, reaching normal levels after 1 year. In the spiny mouse, a precocious species considered to be a reliable animal model to study the fetal development in humans, a limited capacity for endogenous Cr synthesis until late in pregnancy has been observed [5]. It could be supposed that in the first days of life the machinery needed for Cr synthesis does not function to capacity, and the GAA produced is quickly transformed into Cr to replenish the Cr pool in tissues, limiting its loss through the kidneys. Unfortunately, our data does not allow for the possibility of specifying the GA when the Cr metabolic pathway is fully developed.

## 4. Conclusions

Cr likely plays an important role in pregnancy, in particular, when the mother is dependent on an external supply, as for AGAT-d patients. The rapid decline in urinary Cr in our AGAT-d patient between 11 and 21 weeks gestation seems to reflect the needs of the fetus and suggests that an early increase of Cr supplementation would be advantageous. From these data, however, it cannot be stated if a higher Cr dose or an earlier supplementation would have avoided the slight growth delay observed in the fetus.

## Data Availability

Datasets used and /or analysed in the current study are available from the corresponding author upon reasonable request.

## Abbreviations

Cr: Creatine
AGAT-d: Arginine:Glycine Amidino-Transferase deficiency
GAMT: Guanidinoacetate methyltransferase
GA: Gestational age
Crn: Creatinine
w+d: Weeks+ days
GAA: Guanidino acetic acid
CRL: Crown rump length
BPD: Biparietal diameter
HC: Head circumference
AC: Abdominal circumference
FL: Femoral length
